# 2,4,6-Tri­nitro­phenyl 3-chloro­benzoate

**DOI:** 10.1107/S1600536813013792

**Published:** 2013-05-25

**Authors:** Rodolfo Moreno-Fuquen, Fabricio Mosquera, Javier Ellena, C. A. De Simone, Juan C. Tenorio

**Affiliations:** a Departamento de Química, Facultad de Ciencias, Universidad del Valle, Apartado 25360, Santiago de Cali, Colombia; b Instituto de Física de São Carlos, IFSC, Universidade de São Paulo, USP, São Carlos, SP, Brazil

## Abstract

In the title benzoate derivative, C_13_H_6_ClN_3_O_8_, the planes of the benzene rings form a dihedral angle of 73.59 (7)°. The central ester unit forms an angle of 20.38 (12)° with the chloro-substituted benzene ring. In the crystal, mol­ecules are linked by weak C—H⋯O inter­actions, forming helical chains along [101] and [100].

## Related literature

For investigations on reaction kinetics, see: Kirkien-Konasiewicz & Maccoll (1964 ▶); Belousova *et al.* (2000 ▶). For spectroscopic and theoretical studies, see: Ibrahim *et al.* (2011 ▶). For bond-length data, see: Allen *et al.* (1987 ▶). For similar structures, see: Moreno-Fuquen *et al.* (2012*a* ▶,*b* ▶,*c* ▶, 2013 ▶). For hydrogen bonding, see: Nardelli (1995 ▶) and for hydrogen-bond motifs, see: Etter *et al.* (1990 ▶). For a description of the Cambridge Structural Database, see: Allen (2002 ▶).
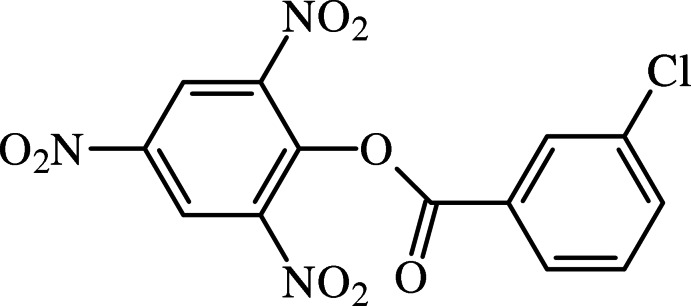


## Experimental

### 

#### Crystal data

C_13_H_6_ClN_3_O_8_
*M* *_r_* = 367.66
Monoclinic, 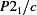

*a* = 11.0633 (4) Å
*b* = 9.6560 (4) Å
*c* = 14.0251 (6) Å
β = 94.009 (2)°
*V* = 1494.60 (10) Å^3^
*Z* = 4
Mo *K*α radiation
μ = 0.31 mm^−1^
*T* = 295 K
0.24 × 0.24 × 0.17 mm

#### Data collection

Nonius KappaCCD diffractometer
16939 measured reflections
3383 independent reflections
2015 reflections with *I* > 2σ(*I*)
*R*_int_ = 0.069

#### Refinement

*R*[*F* ^2^ > 2σ(*F* ^2^)] = 0.051
*wR*(*F* ^2^) = 0.162
*S* = 1.00
3383 reflections
227 parameters
H-atom parameters constrained
Δρ_max_ = 0.32 e Å^−3^
Δρ_min_ = −0.27 e Å^−3^

### 

Data collection: *COLLECT* (Nonius, 2000 ▶); cell refinement: *SCALEPACK* (Otwinowski & Minor, 1997 ▶); data reduction: *DENZO* (Otwinowski & Minor, 1997 ▶) and *SCALEPACK*; program(s) used to solve structure: *SHELXS97* (Sheldrick, 2008 ▶); program(s) used to refine structure: *SHELXL97* (Sheldrick, 2008 ▶); molecular graphics: *ORTEP-3 for Windows* (Farrugia, 2012 ▶) and *Mercury* (Macrae *et al.*, 2006 ▶); software used to prepare material for publication: *WinGX* (Farrugia, 2012 ▶).

## Supplementary Material

Crystal structure: contains datablock(s) I, global. DOI: 10.1107/S1600536813013792/hg5316sup1.cif

Structure factors: contains datablock(s) I. DOI: 10.1107/S1600536813013792/hg5316Isup2.hkl

Supplementary material file. DOI: 10.1107/S1600536813013792/hg5316Isup3.cml

Additional supplementary materials:  crystallographic information; 3D view; checkCIF report

## Figures and Tables

**Table 1:** Hydrogen-bond geometry (Å, °)

*D*—H⋯*A*	*D*—H	H⋯*A*	*D*⋯*A*	*D*—H⋯*A*
C3—H3⋯O3^i^	0.93	2.50	3.167 (3)	128
C11—H11⋯O1^ii^	0.93	2.46	3.263 (3)	145
C13—H13⋯O4^iii^	0.93	2.41	3.312 (3)	165
C10—H10⋯O2^iv^	0.93	2.60	3.278 (3)	131
C5—H5⋯O8^v^	0.93	2.48	3.404 (3)	174

Symmetry codes: (i) 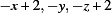; (ii) 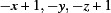; (iii) 
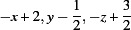
; (iv) 
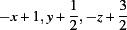
; (v) 
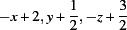
.

**Fig. 1.**
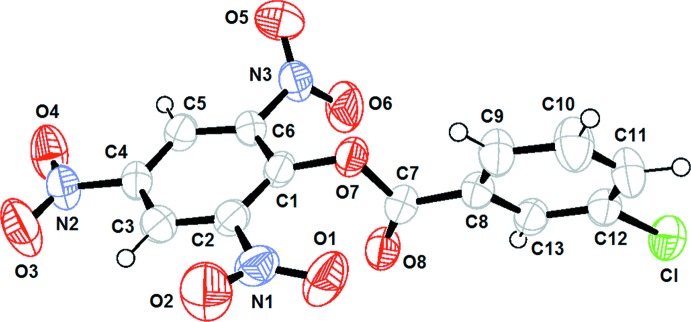
Molecular conformation and atom numbering scheme for the title compound with displacement ellipsoids drawn at the 50% probability level. H atoms are shown as spheres of arbitrary radius.

**Fig. 2.**
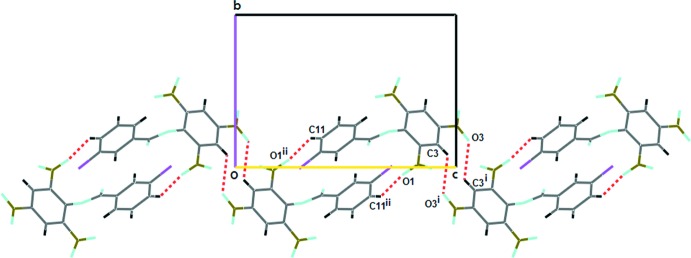
Part of the crystal structure of (I), forming one-dimensional helical chains, along [101]. Symmetry code: (i) -x+2,-y,-z+2; (ii) -x+1,-y,-z+1.

**Fig. 3.**
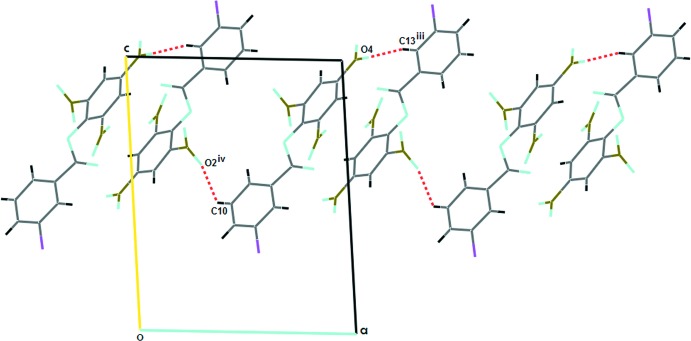
Part of the crystal structure of (I), showing the formation of chains which running along along [100]. Symmetry code: (iii) -x+2,+y-1/2,-z+3/2; (iv) -x+1,+y+1/2,-z+3/2.

## supplementary crystallographic information

### Comment

The title compound, C_13_H_6_ClN_3_O_8_[2,4,6-trinitrophenyl 3-chlorobenzoate] (I), belongs to a group of molecules known as picryl substituted-benzoates (or 2,4,6-trinitrofenil substituted-benzoates). Our research group has been lately investigating about this type of compound, in order to complete the crystallographic information around picryl substituted-benzoates. Also, the structural information can be linked or be useful to explain the results found at investigations of reaction kinetics (Kirkien-Konasiewicz & Maccoll, 1964; Belousova *et al.*, 2000), spectroscopic behavior and theoretical studies (Ibrahim *et al.*, 2011) underwent over this same group of compounds. The molecular structure of (I) is shown in Fig. 1, with a numbering scheme similar to that for TNP4ClBA (Moreno-Fuquen *et al.*, 2013), TNP3MeBA (Moreno-Fuquen *et al.*, 2012*a*), TNP4MeBA(Moreno-Fuquen *et al.*, 2012*b*) and TNPBA (Moreno-Fuquen *et al.*, 2012*c*) in order to simplify structural comparisons. As a general fact, described deeply in previous papers (Moreno-Fuquen *et al.*, 2012*c* and 2013), the structural parameters of substituted picryl benzoates, including (I), show significant differences in the bond distances C1—O7 and C7—O7 if they are compared with analogous distances in other phenyl benzoates reported in the literature (Allen, 2002, Version 5.33). The benzene rings of (I) form a dihedral angle of 73.59 (7)°. The central ester moiety forms an angle of 20.38 (12)° with the chloro-substituted benzene ring to which it is attached and an angle of 86.03 (7)° with the picryl ring. The nitro groups form dihedral angles with the adjacent benzene ring of 59.14 (9)°, 3.6 (2)° and 21.48 (14)° for O1—N1—O2, O3—N2—O4 and O5—N3—O6, respectively. The molecules are packed in a three dimensional network, through weak interactions C—H···O (see Table 1; Nardelli, 1995). Weak C3-H3···O3 and C11-H11···O1 contacts which reinforced each other, allow the molecules to propagate, forming one-dimensional helical chains, along [101]. Both weak contacts form dimers within the structure, as is shown in Fig. 2, and allow the formation of R_2_^2^(10) and R_2_^2^(22) rings respectively (Etter, 1990). In addition to the mentioned interactions other weak C-H···O interactions are observed. Indeed, the C13-H13···O4 and C10-H10···O2 weak contacts, form C(9) and C(12) chains of molecules allowing the crystal to grow also along [100] (see Fig. 3).

### Experimental

The reagents and solvents for the synthesis were obtained from the Aldrich Chemical Co., and were used without additional purification. The title molecule was obtained through a two-step reaction. First the 3-chlorobenzoic acid (0.20g, 0.554 mmol) was refluxed in an excess of thionyl chloride (10 ml) during an hour. Then thionyl chloride was distilled off under reduced pressure to purify the 3-chorobenzoyl chloride obtained as a pale yellow traslucent liquid. The same reaction flask was rearranged and a solution of picric acid (0.12 g, 0.554 mmol) in acetonitrile was added dropwise with constant stirring. The reaction mixture was left to reflux for about an hour. A pale yellow solid was obtained after leaving the solvent to evaporate. The solid was washed with distilled water and cold methanol to eliminate impurities. Crystals of good quality and suitable for single-crystal X-ray diffraction were grown from acetonitrile. IR spectra were recorded on a FT—IR SHIMADZU IR-Affinity-1 spectrophotometer. Pale Yellow crystals; yield 65%; m.p 408 (1)K. IR (KBr) 3092.32 cm^-1^ (aromatic C—H); 1763.27 cm^-1^ (ester C=O); 1615.65 cm^-1^ (C=C); 1546.61 cm^-1^, 1340.90 cm^-1^ (–NO_2_); 1216.82 cm^-1^ (C(=O)—O).

### Refinement

All the hydrogen atoms attached to C atoms were positioned at geometrically idealized positions and treated as riding with C-H= 0.93 Å with *U*_iso_(H) = 1.2 *U*_eq_(C).

### Crystal data

**Table:**

C_13_H_6_ClN_3_O_8_	*F*(000) = 744
*M**_r_* = 367.66	*D*_x_ = 1.634 Mg m^−^^3^
Monoclinic, *P*2_1_/*c*	Melting point: 408(1) K
Hall symbol: -P 2ybc	Mo *K*α radiation, λ = 0.71073 Å
*a* = 11.0633 (4) Å	Cell parameters from 6920 reflections
*b* = 9.6560 (4) Å	θ = 2.6–27.5°
*c* = 14.0251 (6) Å	µ = 0.31 mm^−^^1^
β = 94.009 (2)°	*T* = 295 K
*V* = 1494.60 (10) Å^3^	Block, pale-yellow
*Z* = 4	0.24 × 0.24 × 0.17 mm

### Data collection

**Table:**

Nonius KappaCCD diffractometer	2015 reflections with *I* > 2σ(*I*)
Radiation source: fine-focus sealed tube	*R*_int_ = 0.069
Graphite monochromator	θ_max_ = 27.5°, θ_min_ = 2.6°
CCD rotation images, thick slices scans	*h* = −13→14
16939 measured reflections	*k* = −11→12
3383 independent reflections	*l* = −17→18

### Refinement

**Table:**

Refinement on *F*^2^	Primary atom site location: structure-invariant direct methods
Least-squares matrix: full	Secondary atom site location: difference Fourier map
*R*[*F*^2^ > 2σ(*F*^2^)] = 0.051	Hydrogen site location: inferred from neighbouring sites
*wR*(*F*^2^) = 0.162	H-atom parameters constrained
*S* = 1.00	*w* = 1/[σ^2^(*F*_o_^2^) + (0.0973*P*)^2^] where *P* = (*F*_o_^2^ + 2*F*_c_^2^)/3
3383 reflections	(Δ/σ)_max_ < 0.001
227 parameters	Δρ_max_ = 0.32 e Å^−^^3^
0 restraints	Δρ_min_ = −0.27 e Å^−^^3^

### Special details

**Table:**

Geometry. All esds (except the esd in the dihedral angle between two l.s. planes) are estimated using the full covariance matrix. The cell esds are taken into account individually in the estimation of esds in distances, angles and torsion angles; correlations between esds in cell parameters are only used when they are defined by crystal symmetry. An approximate (isotropic) treatment of cell esds is used for estimating esds involving l.s. planes.
Refinement. Refinement of *F*^2^ against ALL reflections. The weighted *R*-factor *wR* and goodness of fit *S* are based on *F*^2^, conventional *R*-factors *R* are based on *F*, with *F* set to zero for negative *F*^2^. The threshold expression of *F*^2^ > σ(*F*^2^) is used only for calculating *R*-factors(gt) *etc*. and is not relevant to the choice of reflections for refinement. *R*-factors based on *F*^2^ are statistically about twice as large as those based on *F*, and *R*- factors based on ALL data will be even larger.

### Fractional atomic coordinates and isotropic or equivalent isotropic displacement parameters (Å^2^)

**Table:**

	*x*	*y*	*z*	*U*_iso_*/*U*_eq_	
Cl	0.56852 (7)	−0.00919 (7)	0.28977 (5)	0.0682 (3)	
O7	0.71179 (13)	0.23513 (17)	0.69696 (10)	0.0502 (4)	
O8	0.84485 (15)	0.14324 (18)	0.60062 (12)	0.0578 (5)	
C13	0.6511 (2)	0.0995 (2)	0.45876 (16)	0.0471 (5)	
H13	0.7174	0.0411	0.4550	0.057*	
C3	0.8964 (2)	0.1510 (2)	0.91560 (16)	0.0465 (5)	
H3	0.9003	0.0823	0.9622	0.056*	
C1	0.80334 (19)	0.2457 (2)	0.76791 (15)	0.0440 (5)	
N2	1.0634 (2)	0.2709 (2)	1.00374 (16)	0.0597 (6)	
C8	0.6420 (2)	0.1866 (2)	0.53711 (16)	0.0464 (5)	
C5	0.9641 (2)	0.3704 (2)	0.85707 (17)	0.0499 (6)	
H5	1.0150	0.4469	0.8641	0.060*	
N1	0.7383 (2)	0.0189 (2)	0.82752 (16)	0.0566 (5)	
C7	0.7442 (2)	0.1841 (2)	0.61029 (16)	0.0474 (5)	
C2	0.81502 (19)	0.1428 (2)	0.83739 (16)	0.0441 (5)	
C6	0.8789 (2)	0.3605 (2)	0.78057 (16)	0.0470 (5)	
O3	1.07187 (19)	0.17305 (19)	1.05824 (15)	0.0776 (6)	
C12	0.5594 (2)	0.1017 (2)	0.38669 (16)	0.0503 (6)	
O6	0.8224 (2)	0.4610 (2)	0.63360 (14)	0.0802 (6)	
C4	0.9719 (2)	0.2641 (2)	0.92272 (16)	0.0469 (5)	
N3	0.8689 (2)	0.4788 (2)	0.71367 (16)	0.0587 (6)	
O1	0.7462 (2)	−0.0505 (2)	0.75600 (16)	0.0866 (7)	
O5	0.9087 (2)	0.5889 (2)	0.74352 (17)	0.0942 (8)	
C9	0.5427 (2)	0.2730 (3)	0.54277 (18)	0.0579 (6)	
H9	0.5360	0.3292	0.5960	0.069*	
O2	0.6754 (2)	−0.0070 (2)	0.89145 (18)	0.0891 (7)	
O4	1.1242 (2)	0.3733 (2)	1.01268 (17)	0.1025 (8)	
C11	0.4618 (2)	0.1893 (3)	0.3903 (2)	0.0652 (7)	
H11	0.4016	0.1910	0.3406	0.078*	
C10	0.4542 (3)	0.2748 (3)	0.4686 (2)	0.0697 (8)	
H10	0.3884	0.3343	0.4713	0.084*	

### Atomic displacement parameters (Å^2^)

**Table:**

	*U*^11^	*U*^22^	*U*^33^	*U*^12^	*U*^13^	*U*^23^
Cl	0.0769 (5)	0.0784 (5)	0.0485 (4)	−0.0108 (3)	−0.0009 (3)	−0.0157 (3)
O7	0.0480 (9)	0.0652 (10)	0.0368 (9)	0.0050 (7)	−0.0020 (7)	−0.0033 (7)
O8	0.0538 (10)	0.0734 (11)	0.0453 (10)	0.0105 (8)	−0.0036 (8)	−0.0074 (8)
C13	0.0495 (12)	0.0508 (13)	0.0408 (12)	0.0009 (10)	0.0008 (10)	0.0015 (10)
C3	0.0558 (13)	0.0431 (12)	0.0403 (12)	−0.0014 (10)	0.0003 (10)	0.0003 (9)
C1	0.0440 (12)	0.0551 (13)	0.0326 (11)	0.0029 (10)	0.0007 (9)	−0.0035 (9)
N2	0.0611 (13)	0.0580 (13)	0.0574 (14)	−0.0079 (10)	−0.0132 (10)	−0.0038 (10)
C8	0.0477 (13)	0.0512 (13)	0.0396 (12)	−0.0023 (10)	−0.0010 (10)	0.0024 (10)
C5	0.0533 (13)	0.0472 (12)	0.0491 (14)	−0.0072 (10)	0.0042 (11)	−0.0023 (10)
N1	0.0571 (12)	0.0555 (12)	0.0553 (13)	−0.0098 (9)	−0.0103 (10)	0.0040 (10)
C7	0.0533 (14)	0.0499 (13)	0.0385 (12)	0.0029 (10)	−0.0002 (10)	−0.0027 (10)
C2	0.0453 (12)	0.0461 (12)	0.0405 (12)	−0.0057 (9)	0.0004 (10)	−0.0036 (9)
C6	0.0522 (13)	0.0498 (13)	0.0393 (12)	−0.0007 (10)	0.0053 (10)	0.0013 (9)
O3	0.0935 (15)	0.0600 (12)	0.0733 (14)	−0.0002 (10)	−0.0353 (11)	0.0072 (10)
C12	0.0537 (14)	0.0548 (14)	0.0418 (13)	−0.0087 (10)	−0.0004 (10)	−0.0006 (10)
O6	0.1168 (18)	0.0773 (13)	0.0449 (11)	0.0013 (11)	−0.0049 (11)	0.0127 (9)
C4	0.0481 (12)	0.0509 (13)	0.0408 (12)	−0.0020 (10)	−0.0044 (10)	−0.0060 (10)
N3	0.0681 (14)	0.0589 (14)	0.0496 (13)	−0.0026 (10)	0.0087 (11)	0.0087 (10)
O1	0.1140 (18)	0.0723 (13)	0.0715 (15)	−0.0338 (12)	−0.0082 (13)	−0.0195 (11)
O5	0.130 (2)	0.0646 (13)	0.0845 (16)	−0.0310 (13)	−0.0140 (14)	0.0210 (12)
C9	0.0560 (14)	0.0650 (15)	0.0510 (15)	0.0085 (12)	−0.0076 (12)	−0.0121 (12)
O2	0.0872 (15)	0.0940 (16)	0.0878 (17)	−0.0358 (12)	0.0189 (13)	0.0113 (12)
O4	0.1119 (18)	0.0926 (16)	0.0960 (18)	−0.0533 (14)	−0.0425 (14)	0.0164 (13)
C11	0.0568 (15)	0.0773 (18)	0.0582 (17)	0.0004 (13)	−0.0189 (12)	−0.0063 (14)
C10	0.0617 (16)	0.0768 (18)	0.0680 (19)	0.0194 (14)	−0.0144 (14)	−0.0113 (15)

### Geometric parameters (Å, º)

**Table:**

Cl—C12	1.739 (2)	C8—C7	1.473 (3)
O7—C1	1.373 (3)	C5—C4	1.377 (3)
O7—C7	1.382 (3)	C5—C6	1.381 (3)
O8—C7	1.198 (3)	C5—H5	0.9300
C13—C12	1.382 (3)	N1—O2	1.199 (3)
C13—C8	1.393 (3)	N1—O1	1.215 (3)
C13—H13	0.9300	N1—C2	1.468 (3)
C3—C2	1.372 (3)	C6—N3	1.478 (3)
C3—C4	1.375 (3)	C12—C11	1.375 (3)
C3—H3	0.9300	O6—N3	1.214 (3)
C1—C2	1.392 (3)	N3—O5	1.213 (3)
C1—C6	1.392 (3)	C9—C10	1.379 (4)
N2—O4	1.198 (3)	C9—H9	0.9300
N2—O3	1.215 (3)	C11—C10	1.380 (4)
N2—C4	1.469 (3)	C11—H11	0.9300
C8—C9	1.386 (3)	C10—H10	0.9300
			
C1—O7—C7	116.14 (17)	C3—C2—C1	122.9 (2)
C12—C13—C8	118.5 (2)	C3—C2—N1	117.73 (19)
C12—C13—H13	120.7	C1—C2—N1	119.42 (19)
C8—C13—H13	120.7	C5—C6—C1	121.7 (2)
C2—C3—C4	117.7 (2)	C5—C6—N3	117.2 (2)
C2—C3—H3	121.1	C1—C6—N3	121.1 (2)
C4—C3—H3	121.1	C11—C12—C13	121.4 (2)
O7—C1—C2	118.82 (19)	C11—C12—Cl	119.78 (18)
O7—C1—C6	123.9 (2)	C13—C12—Cl	118.81 (19)
C2—C1—C6	117.0 (2)	C3—C4—C5	122.3 (2)
O4—N2—O3	123.9 (2)	C3—C4—N2	118.3 (2)
O4—N2—C4	118.0 (2)	C5—C4—N2	119.4 (2)
O3—N2—C4	118.1 (2)	O5—N3—O6	124.3 (2)
C9—C8—C13	120.7 (2)	O5—N3—C6	116.7 (2)
C9—C8—C7	122.8 (2)	O6—N3—C6	119.0 (2)
C13—C8—C7	116.5 (2)	C10—C9—C8	119.2 (2)
C4—C5—C6	118.4 (2)	C10—C9—H9	120.4
C4—C5—H5	120.8	C8—C9—H9	120.4
C6—C5—H5	120.8	C12—C11—C10	119.3 (2)
O2—N1—O1	125.6 (2)	C12—C11—H11	120.4
O2—N1—C2	117.5 (2)	C10—C11—H11	120.4
O1—N1—C2	116.9 (2)	C9—C10—C11	120.9 (2)
O8—C7—O7	121.1 (2)	C9—C10—H10	119.6
O8—C7—C8	126.9 (2)	C11—C10—H10	119.6
O7—C7—C8	111.96 (19)		
			
C7—O7—C1—C2	−99.7 (2)	C2—C1—C6—C5	1.8 (3)
C7—O7—C1—C6	86.9 (3)	O7—C1—C6—N3	−3.1 (3)
C12—C13—C8—C9	0.3 (3)	C2—C1—C6—N3	−176.59 (19)
C12—C13—C8—C7	−177.8 (2)	C8—C13—C12—C11	1.2 (3)
C1—O7—C7—O8	4.8 (3)	C8—C13—C12—Cl	−178.73 (16)
C1—O7—C7—C8	−175.72 (18)	C2—C3—C4—C5	3.1 (3)
C9—C8—C7—O8	−158.6 (3)	C2—C3—C4—N2	−177.1 (2)
C13—C8—C7—O8	19.5 (4)	C6—C5—C4—C3	−1.4 (3)
C9—C8—C7—O7	21.9 (3)	C6—C5—C4—N2	178.8 (2)
C13—C8—C7—O7	−159.95 (19)	O4—N2—C4—C3	−175.8 (3)
C4—C3—C2—C1	−2.4 (3)	O3—N2—C4—C3	3.4 (3)
C4—C3—C2—N1	177.2 (2)	O4—N2—C4—C5	4.0 (4)
O7—C1—C2—C3	−173.9 (2)	O3—N2—C4—C5	−176.8 (2)
C6—C1—C2—C3	0.0 (3)	C5—C6—N3—O5	−20.4 (3)
O7—C1—C2—N1	6.5 (3)	C1—C6—N3—O5	158.0 (2)
C6—C1—C2—N1	−179.6 (2)	C5—C6—N3—O6	159.5 (2)
O2—N1—C2—C3	57.8 (3)	C1—C6—N3—O6	−22.1 (3)
O1—N1—C2—C3	−120.5 (2)	C13—C8—C9—C10	−1.8 (4)
O2—N1—C2—C1	−122.5 (3)	C7—C8—C9—C10	176.3 (2)
O1—N1—C2—C1	59.2 (3)	C13—C12—C11—C10	−1.3 (4)
C4—C5—C6—C1	−1.1 (3)	Cl—C12—C11—C10	178.6 (2)
C4—C5—C6—N3	177.3 (2)	C8—C9—C10—C11	1.6 (4)
O7—C1—C6—C5	175.3 (2)	C12—C11—C10—C9	−0.1 (4)

### Hydrogen-bond geometry (Å, º)

**Table:**

*D*—H···*A*	*D*—H	H···*A*	*D*···*A*	*D*—H···*A*
C3—H3···O3^i^	0.93	2.50	3.167 (3)	128
C11—H11···O1^ii^	0.93	2.46	3.263 (3)	145
C13—H13···O4^iii^	0.93	2.41	3.312 (3)	165
C10—H10···O2^iv^	0.93	2.60	3.278 (3)	131
C5—H5···O8^v^	0.93	2.48	3.404 (3)	174

Symmetry codes: (i) −*x*+2, −*y*, −*z*+2; (ii) −*x*+1, −*y*, −*z*+1; (iii) −*x*+2, *y*−1/2, −*z*+3/2; (iv) −*x*+1, *y*+1/2, −*z*+3/2; (v) −*x*+2, *y*+1/2, −*z*+3/2.
